# Retraction Note: Superparamagnetic iron oxide nanoparticles conjugated with Aβ oligomer-specific ScFv antibody and class A scavenger receptor activator show therapeutic potentials for alzheimer’s disease

**DOI:** 10.1186/s12951-025-03880-3

**Published:** 2025-11-25

**Authors:** Xiao-ge Liu, Lun Zhang, Shuai Lu, Dong-qun Liu, Ya-ru Huang, Jie Zhu, Wei-wei Zhou, Xiao-lin Yu, Rui-tian Liu

**Affiliations:** 1 State Key Laboratory of Biochemical Engineering, Institute of Process Engineering, Chinese Academy of Sciences, Beijing, 100190 China; 2 School of Chemical Engineering, University of Chinese Academy of Sciences, Beijing, 100049 China

**Retraction Note: Journal of Nanobiotechnology (2020) 18:160**

10.1186/s12951-020-00723-1

The Editor-in-Chief has retracted this article. After publication, image integrity concerns were raised. Specifically, in Fig. 4b, Cortex AD-W20-SPIONs and Hippocampus AD-SPIONs appear to overlap. Figure 5a AD-SPIONs and Fig. 5d AD-W20-SPIONs appear to overlap with frameshift. The authors were unable to provide the original images for their proposed correction upon request. The Editor-in-Chief has lost confidence in the data and conclusions of this article.

Author Ruitian Liu has not stated explicitly if they agree or disagree with the retraction. All other authors did not respond to correspondence from the publisher regarding this retraction.

